# Depression and Anxiety Outcomes Among Young Adults Who Self-Reported Experiencing Commercial Sexual Exploitation in Adolescence

**DOI:** 10.3390/ijerph22071062

**Published:** 2025-07-02

**Authors:** Sarah M. Godoy, Adam R. Englert, Nofar Mazursky, Luisa Prout, William J. Hall

**Affiliations:** 1 Silver School of Social Work, New York University, New York, NY 10003, USA; ltp2027@nyu.edu; 2 School of Social Work, University of North Carolina at Chapel Hill, Chapel Hill, NC 27517, USA; arengler@unc.edu (A.R.E.); wjhall@email.unc.edu (W.J.H.); 3 Marron Institute of Urban Management, New York University, Brooklyn, NY 11201, USA; nm4567@nyu.edu; 4 Center for Urban Science and Progress, Tandon School of Engineering, New York University, Brooklyn, NY 11201, USA

**Keywords:** human trafficking, sex trafficking, mental health, comorbidity, family dynamics, family health, add health, nationally representative data

## Abstract

The commercial sexual exploitation (CSE) of children is a distinct form of sexual trauma, resulting in immediate mental health issues. Few studies explore associations between family-level factors in adolescence and health outcomes in adulthood among this population. Utilizing a nationally representative dataset, we explored differences and associations between mental health outcomes and domains of the Family Health Development framework among respondents who self-reported CSE (*N* = 502; mean age = 15.03, *SD* = 1.34; 67% male; 50% white). We conducted ordinary least squares and binary logistic regressions using a hierarchical approach to analyze the CES-D depression scale, anxious personality scale, and self-reported diagnoses of depression and anxiety/panic disorder. At Wave IV, when participants were aged 24–32, 20% of participants reported ever having a diagnosis of depression, and 12% reported ever having an anxiety/panic disorder diagnosis. Family receipt of public assistance during adolescence significantly predicted depression and anxiety symptoms in adulthood, highlighting associations between family structure and mental health. Gender and race significantly predicted anxiety symptoms and having a diagnosis of depression and anxiety/panic disorder. Findings underscore the need for targeted training and comprehensive health screenings for providers to better understand and address the long-term mental health needs of CSE-impacted groups.

## 1. Introduction

The commercial sexual exploitation (CSE) of children is a human rights violation and grave form of sexual victimization that has immediate, negative health outcomes. Exploited children often endure concurrent forms of physical, sexual, and psychological violence including coercive tactics such as rape, lack of autonomy and restricted movement, and constant threats [1,2,3,4]. As a result, these children frequently experience mental health issues, including depression, anxiety, and posttraumatic stress disorder (PTSD) [3,5].

In the United States, the Trafficking Victims Protection Act (TVPA 2000, P.L. 106–386) defines child sex trafficking, commonly referred to as the CSE of children, as the exchange of sexual acts for anything of value (e.g., goods, services, drugs, or money) by a minor under the age of 18. Therefore, any minor involved in a commercial sex act—regardless of the presence of force, fraud, or coercion—is classified as sex trafficked, as they cannot legally consent to trade sex. The prevalence of minors experiencing CSE is largely unknown [6] as the covert nature of this crime makes it difficult to estimate its scope. Individuals who experience CSE are often under-identified, especially marginalized groups largely due to a lack of standardized systems or databases for collecting information on CSE-impacted populations [7].

Research indicates that CSE-impacted children are exploited by family members, caregivers’ partners, romantic partners, peers, friends, acquaintances, and strangers [8]. These children often intersect with multiple service systems, including child welfare, juvenile legal, education, and healthcare [8,9,10]. Thus, multiple sectors of society bear responsibility for addressing issues affecting CSE-impacted populations. In order to adequately serve these individuals, it is imperative that we have a better understanding of the long-term negative mental health outcomes associated with CSE.

## 2. Background

### 2.1. Mental Health Issues Among CSE-Impacted Populations

CSE-impacted adolescents and young adults frequently experience mental health challenges, with depression and anxiety among the most commonly reported diagnoses [5,11,12,13,14]. Although comprehensive prevalence estimates are lacking, existing data from relatively small samples offer valuable insights into the mental health burden within this population. For instance, studies report that 78% (*N* = 23) of adolescent girls and boys in a community-based sample, 73% (*n* = 265) of adolescents in the juvenile legal system who had contact with a mental health professional, and 52% (*N* = 128) of adolescent girls in the juvenile legal system were diagnosed with at least one mental health condition [5,12]. Among adolescents in the juvenile legal system, the most prevalent diagnoses were depression (68%), sleep disorder (52%), mood disorder (50%), and disruptive behavior disorder (40%) [5].

Research indicates that CSE has an independent, significant effect on PTSD, anxiety, and depression outcomes, even when accounting for prior childhood abuse [15]. Duration of time since exploitation has also been identified as an important factor influencing mental health among CSE-impacted individuals. Studies show that the extent of time since CSE exposure is associated with varying levels of anxiety and depression [15]. These studies provide important insights into how CSE impacts mental health. Still, less is known about the long-term mental health outcomes of individuals who experience CSE during adolescence [16,17] or how family health dynamics and factors may be associated with depression and anxiety.

Adolescents impacted by CSE experience heightened vulnerabilities to immediate and long-term adverse mental health issues, compared to non-CSE exposed counterparts [11,12,14,18]. Indeed, studies consistently show that CSE-impacted adolescents experience significantly higher rates of attention-deficit/hyperactivity disorder (ADHD), anxiety, bipolar disorder, conduct disorder, and depression [12,14]. One study found that among adolescents in the juvenile legal system, those who experienced CSE were nearly 10 times more likely to be diagnosed with clinical depression compared to those who had not experienced CSE [12]. Compared to other high-risk groups without CSE exposure (i.e., children who run away from home, those in foster care, and children in the juvenile legal system), CSE-impacted adolescents had significantly worse mental health outcomes, including higher rates of ADHD (52%), depression (46%), bipolar disorder (27%), anxiety (20%), conduct disorder (20%), oppositional defiant disorder (20%), and PTSD (20%) [14]. CSE-impacted young men experience higher rates of depression compared to peers without CSE exposure (22% vs. 13%) [16]. These findings underscore heightened mental health challenges faced by CSE-impacted groups and the increased likelihood of comorbidity–the presence of multiple mental health diagnoses—which further complicates treatment and recovery.

Comorbidity is an additional serious issue for CSE-impacted adolescents. Studies report that between 65% and 88% of their samples have experienced comorbid conditions, including a combination of depression, anxiety, and PTSD [5,11,14,19]. Moreover, the duration of CSE exposure has been shown to influence the severity of co-occurring mental health issues. For instance, one study suggests that longer exposure to CSE was associated with higher levels of anxiety and depression in women and girls [15]. Despite this knowledge, we know less about mental health issues, including comorbidity experienced in early adulthood for those commercially sexually exploited during adolescence.

### 2.2. Guiding Theoretical Framework

Family Health Development is an integrative model that serves as a guiding theory, positing that families are central to child and adolescent developmental health outcomes [20]. This framework focuses on four domains of family well-being: (1) family structure; (2) family process; (3) cognitions; and (4) health behaviors. These domains of family health depict interactions between social, behavioral, cultural, and economic contexts that can modify individual functioning and influence symptomatic risks of mental health challenges [21]. Family Health Development provides a heuristic life course model and has become an important area of research to explain how health trajectories develop over an individual’s lifespan, and how this knowledge can inform new approaches to policy and research [20,21]. To our knowledge, this theory has not been applied to populations affected by human trafficking. Therefore, the Family Health Development framework is utilized as the guiding theoretical approach to examine individual- and family-level factors associated with mental health outcomes among CSE-impacted individuals.

#### 2.2.1. Family Structures and Processes Domains

The Family Health Development emphasizes the foundational role of family structures and processes in shaping health trajectories. These influences may be particularly pronounced among CSE-impacted populations, where instability and disruption within family environments may heighten their vulnerability to mental health challenges. Indeed, research suggests that family structures and processes in childhood are critical to shaping mental health outcomes in adulthood, including depression and anxiety [22,23]. In the general population, research indicates that adults who grew up in single-parent households experience higher odds of depression diagnoses compared to those raised in married, two-parent households [24]. Family structure may have a greater impact on adolescent girls than their male counterparts, as a gender gap in self-reported mental health issues emerge and widen for those from single-parent and blended families, compared to those in two-parent households [25]. One study shows that white individuals with married parents at age 15 have lower levels of anxiety at age 33; however, this protective factor is smaller among their African American counterparts [26]. Additionally, financial components of family structure, such as growing up in a family receiving public assistance, is associated with higher depression scores in the general population [27].

Early human trafficking research suggests that stronger family connectedness and improved relationships between CSE-impacted young people and their parents are associated with greater self-esteem, emotional resilience, and lower levels of emotional distress [28]. Family processes and quality of parent–child relationships are important components of family structure. Family processes have been identified as sources of individual well-being as numerous components of the family system in which the individual is embedded are shown to significantly impact mental health outcomes like depression [29]. Given the established links between family structure, family processes, and mental health outcomes in the general population, it is crucial to consider how these factors manifest within CSE-impacted populations, specifically related to depression and anxiety.

#### 2.2.2. Cognitions Domain

The Family Health Development framework identifies cognition as a key influence on health, highlighting how internal thought patterns and interpretations inform individual and family well-being across the life course. Cognition is a widely used term with varied definitions that tend to encompass constructs of individual thinking, memory, perception, problem solving, intelligence, reasoning, language, and creativity [30]. Cognitions are critical components of family health, as perspectives of one’s self and others, health knowledge and literacy, and health-related values and priorities shape aspects of health development [20].

Vocabulary comprehension is a measure that highly correlates with overall cognitive function [31]. When individuals grow up in healthy environments, they often reflect an upbringing conducive to developing basic and complex reading skills and general cognitive ability [31]. Childhood cognitive ability demonstrates protective effects against both anxiety and depression symptoms [32]. This protective effect attenuates stressors experienced in adolescence that contribute to anxious and depressive symptoms, regardless of gender [33]. On the other hand, early childhood sexual abuse negatively impacts individual cognitive abilities like scholastic aptitude and language skills [34]. Research examining the neuropsychological effects of CSE during adolescence shows reduced intelligence scores, slower performance, and increased errors on tasks for working memory [35]. The potential consequences of CSE appear especially salient during adolescence [33,36], and cognitive ability challenges at this life stage can cascade into later adulthood.

While the impact of cognitive development and health literacy on mental health outcomes is established in the literature, a notable gap persists regarding how these factors intersect within CSE-impacted groups. Additionally, research demonstrates associations between poor health literacy and depression [37]; however, this association is captured using broad measures of health literacy. Indeed, we know less about how health knowledge related to specific health-related behaviors, such as physical fitness and substance use, influences mental health symptoms. Similarly, the relationship between poor health literacy and depression has been documented among those with problematic substance and alcohol use [38]. Yet, research examining associations between health literacy and depression or anxiety among CSE-impacted groups is sparse.

#### 2.2.3. Health-Related Behaviors Domain

The Family Health Development framework highlights high-behaviors as a core domain influencing immediate and long-term health outcomes. Health-related behaviors, such as seeking medical and mental health treatment, engaging in physical activity, and substance use, are linked to child health and development as well as health outcomes in adulthood [20], including psychological well-being [39]. Research indicates that family health values and priorities shape family health decisions, including willingness to invest resources in health treatment or services [40]. In general, placing value on health is associated with prompting positive change in health and help-seeking practices [41]. This change is linked to increased positive outcomes, such as life enjoyment, connectedness, and quality of life [42], and has the potential to decrease negative health outcomes.

Research has found that CSE-impacted adolescents and young adults frequently endure serious injuries and illnesses as a result of the exploitation [2,4,43]. While studies indicate that, at times, these individuals are able to engage in emergency, physical, reproductive, and mental healthcare, CSE-impacted adolescents and young adults are also frequently unable to access care [2,43,44,45]. Those who experience CSE may be unable to access medical treatment due to trafficker control, lack of transportation, or fear of criminalization and stigma, among other reasons [2]. Despite these barriers, less is known about how engaging in mental health treatment or requiring but not seeking medical treatment affects long-term mental health outcomes among CSE-impacted adolescents.

While research regarding nutritional status and physical activity among CSE-impacted individuals is limited [12,46], these factors have been studied to protect against negative mental health outcomes in the general population. For example, exercise is a well-established behavior that protects against depressive symptoms. Studies have shown regular exercise can provide a sustained positive effect on depression during adolescence [47]. It is well-documented that CSE-impacted adolescents are burdened by higher rates of substance use than non-exploited counterparts, which is associated with mental health issues [5,12,48,49]. Still, we know little about the associations between substance use and depression or anxiety outcomes among these adolescents. Therefore, understanding the role of health behaviors among CSE-impacted adolescents may be crucial for understanding their health outcomes in young adulthood.

### 2.3. Current Study

This study aimed to understand how domains of the Family Health Development framework (i.e., family structure, family processes, cognitions, and health behaviors) help predict negative mental health outcomes (i.e., depression and anxiety) among young adults with a history of CSE. The research questions explored in this study are as follows: Among young adults who self-report experiencing CSE in adolescence, (1) what are their average depression and anxious personality scores? (2) What proportion had a depression and/or anxiety/panic disorder diagnosis? (3) Are there differences and associations in mental health outcomes (i.e., depression scores, anxious personality scores) based on family structure, family process, cognitions, health behaviors, and demographic characteristics (i.e., race/ethnicity and gender identity)? (4) Are there differences and associations in ever being diagnosed with depression or anxiety/panic disorder based on family structure, family process, cognitions, health behaviors, and demographic characteristics?

## 3. Materials and Methods

### 3.1. Study Design and Data

This study used data from the National Longitudinal Study of Adolescent to Adult Health (Add Health) in-home interviews with adolescents and parents at Waves I, II, and IV [50,51]. Add Health used a school-based design to include 80 high schools and a paired feeder school (e.g., middle school) to administer in-school questionaries to more than 90,000 students [51]. Using school rosters, a second level of sampling was then used to conduct 90 min in-home interviews with adolescents and a 30 min interview with one of their parents [51]. Add Health was designed by a multidisciplinary team from biomedical, behavioral, and social sciences to examine causes of health and health behavior [51]. Wave I data were collected from 1994 to 1995 and included a nationally representative sample of over 20,000 adolescents in grades 7 through 12. Wave II data were collected in 1996. Wave IV data were collected from 2008 to 2009 and included more than 15,000 respondents aged 24 to 32 from Wave I. In-home parent surveys were collected at Wave I and included over 17,000 respondents. Carolina Population Center provided access to restricted data and the Institutional Review Board at University of North Carolina at Chapel Hill (IRB # 22-1687) approved this study.

### 3.2. Sample

The analytic sample was restricted to adolescents who self-reported CSE as a minor based on two items collected at Waves I and II (*N* = 502). At Wave I, participants were asked, “Have you ever given someone sex in exchange for drugs or money?” Those who responded “yes” to this question and were below age 18 were included in the sample. At Wave II, participants were asked, “Since [last interview], how many times have you given someone sex in exchange for drugs or money?” Those who responded one or more times and were below age 18 were included in the sample. Therefore, only adolescents below the age of 18 who indicated they had exchanged sex for money or drugs at Waves I and/or II were included in this study. See Table 1 for an overview of sample characteristics.

### 3.3. Measures

#### 3.3.1. Dependent Variables

Four dependent variables reflected depression and anxiety outcomes at Wave IV.

##### Depression Scale

Depression was measured using five items derived from the Center for Epidemiologic Studies Depression Scale (CES-D) at Wave IV. These items asked participants how often the following was true during the past seven days: (a) had trouble keeping their mind on what they were doing; (b) felt depressed; (c) felt sad; (d) could not shake off the blues; and (e) were easily bothered. Responses were captured using a 4-point scale (0 = never or rarely; 3 = most of the time or all of the time). The sum of the five items was calculated to yield a continuous measure ranging from 0 to 15, with higher scores indicating the highest frequency and severity of depressive symptoms. Cronbach’s alpha reliability was 0.80 for depression items, indicating good internal consistency reliability.

##### Depression Diagnosis

Depression diagnosis was measured by two items that assessed if participants received a depression diagnosis or felt depressed at Wave IV. One item asked: “Has a doctor, nurse or other healthcare provider ever told you that you have or had: depression?” Responses were dichotomous (0 = no; 1 = yes). A second item asked: “How often was the following true during the past seven days? You felt depressed.” Responses were captured using a 4-point scale (0 = never or rarely; 3 = most of the time or all of the time) and dichotomized (0 = no; 1 = yes) to indicate if they had or had not felt depressed in the prior seven days. These items were combined to indicate a depression diagnosis (0 = no; 1 = yes).

##### Anxious Personality Scale

The Anxious Personality Scale assessed self-reported levels of anxiety using four items measured at Wave IV. These items asked participants how much they agreed with the following: (a) “I worry about things” (reverse-coded); (b) “I am not easily bothered by things”; (c) “I get stressed out easily” (reverse-coded); and (d) “I don’t worry about things that have already happened”. Responses were captured using a 5-point scale (1 = strongly agree; 5 = strongly disagree). The sum of the four items was calculated to yield a continuous measure ranging from 4 to 20, with higher scores indicating higher frequency and severity of anxious personality symptoms. Cronbach’s alpha reliability was 0.65 for these items, which is minimally acceptable [52].

##### Anxiety or Panic Disorder

One item assessed anxiety or panic disorder measured at Wave IV. Participants were asked: Has a doctor, nurse or other healthcare provider ever told you that you have or had: anxiety or panic disorder? Responses were dichotomous (0 = no; 1 = yes).

#### 3.3.2. Independent Variables

Independent variables reflected the four health domains of the Family Health Development theory: family structure; family processes; cognitions; and health-related behaviors.

##### Domain 1: Family Structure

Living in a two-parent household was measured by one item (1 = two biological parents; 8 = surrogate non-biological parent) at Wave I [53]. Responses were dichotomized (0 = no; 1 = yes) to indicate if adolescents had or had not resided in a two-parent (i.e., biological, step, adoptive) household. Family receiving public assistance was measured by one item at Wave I. Parent responses were dichotomous (0 = no; 1 = yes), indicating if they had or had not received public assistance.

##### Domain 2: Family Processes

Family belonging was assessed using four items measured at Wave I. Participants were asked: (a) “How much do you feel your family understands you?”; (b) “How much do you feel you want to leave home?” (reverse-coded); (c) “How much do you feel you and your family have fun together?” and (d) “To what extent do you feel your family pays attention to you?” Responses were captured using a 5-point scale (1 = very little; 5 = very much) and coded such that higher values indicated higher levels of family belonging. Responses were averaged and standardized, so that the mean was set to zero and the standard deviation to one. Family belonging items possessed adequate internal consistency reliability (α = 0.76).

Mother–child and father–child relationship quality was assessed using five items [54] measured at Wave I. Participants were asked: (a) how close they feel to their mother/father; (b) how much do you think mother/father cares about you; (c) most of the time, their mother/father is warm and loving toward them (reverse-coded); (d) if they were satisfied with the way their mother/father communicates with them (reverse-coded); and (e) overall, if they were satisfied with their relationship with their mother/father (reverse-coded). Responses were captured using a 5-point scale (1 = very little; 5 = very much) for the first two items and a 5-point scale (1 = strongly disagree; 5 = strongly agree) for the remaining three items. Responses were coded such that higher values indicated higher levels of mother/father–child relationship quality. Responses were averaged and standardized. Mother–child relationship quality items possessed good internal consistency reliability (α = 0.84), and father–child relationship quality items possessed good internal consistency reliability (α = 0.87).

##### Domain 3: Cognitions

Cognitive ability was assessed using the Add Health Picture Vocabulary Test at Wave I [55]. The scores were standardized to be expressed as an intelligence quotient (IQ) metric [56,57]. Health knowledge and literacy was measured at Wave I using four items that assessed if participants received health education about exercise and drug abuse in school. Participants were asked if they learned about the following in a class at school: (a) the importance of exercise; and (b) drug abuse. Responses were dichotomous (0 = no; 1 = yes).

##### Domain 4: Health-Related Behaviors

Health behaviors were assessed using five items that captured receipt of medical treatment, receipt of mental health treatment, engaging in exercise, trouble relaxing, and substance use measured at Wave I. Receipt of health treatment was captured with the following two items: “Has there been any time over the past year when you thought you should get medical care, but you did not?” and “In the past year, have you received psychological or emotional counseling?” Responses were dichotomous (0 = no; 1 = yes). Engaging in exercise was assessed using the following item: “During the past week, how many times did you exercise, such as jogging, walking, karate, jumping rope, gymnastics or dancing?” Responses were captured using a 3-point scale (0 = not at all; 3 = 5x or more) and, using a cut-off score of 1, dichotomized (0 = no; 1 = yes). Trouble relaxing was assessed using the following item: “In the past 12 months, how often have you had trouble relaxing?” Responses were captured using a 4-point scale (0 = never; 4 = every day) and, using a cut-off score of 1, were dichotomized (0 = no; 1 = yes).

Substance use was assessed using five items that captured use of alcohol, marijuana, cocaine, inhalants, and other drugs measured at Wave I. To capture alcohol, participants were asked: “Have you had a drink of beer, wine, or liquor—not just a sip or taste of someone else’s drink—more than 2 or 3 times in your life?” Responses were dichotomous (0 = no; 1 = yes). To capture marijuana, participants were asked: “How old were you when you tried marijuana for the first time?” To capture, other drug use participants were asked their age the first time they tried: (a) marijuana; (b) any kind of cocaine, including powder, freebase, or crack cocaine; (c) inhalants, such as glue or solvents; and (d) any other type of illegal drug, such as LSD, PCP, ecstasy, mushrooms, speed, ice, heroin, or pills, without a doctor’s prescription. Responses captured if they never tried the substance to 18 years old (0 = never; 18 = 18 years old). Using a cut-off score of 1, all substance use items were dichotomized (0 = no; 1 = yes), and combined to capture if participants had ever used any substances by Wave I.

#### 3.3.3. Control Variables

The following control variables were included: gender (0 = male; 1 = female) captured at Wave I; and race (1 = white; 2 = Black; 3 = Latinx, American Indian or Native American, and Asian or Pacific Islander) captured at Wave I. Depression scores, using the 5-item CES-D scale, were measured at Wave I to control for depression during adolescence. Anxiety was not captured at Wave I, and therefore was not controlled.

### 3.4. Data Analysis

A priori power analysis was conducted for multiple linear regression (fixed model, R2 increase) using G*Power (version 3.1.9.6). Results for the multiple linear regression indicated that the required sample size to achieve 80% power for detecting a small-to-medium size effect (*f*^2^ = 0.06) at a significance level of alpha = 0.05 with 17 covariates was *N* = 325. Therefore, our smallest analytic sample size (*N* = 350) was deemed sufficient. Statistical assumptions, including linearity, normality of the residuals, multicollinearity, homoscedasticity, and influential outliers, were checked. To examine associations between predictor variables, we used spearman’s rank correlation coefficient. There were no issues with multicollinearity or influential outliers.

Multiple regression models were conducted, including ordinary least squares (OLS) and binary logistic regressions using a hierarchical approach. OLS was used to examine if predictor variables significantly increased the predictability of depression or anxiety. Binary logistic regression models were conducted to examine ever being diagnosed with depression and anxiety/panic disorder, respectively. Using a hierarchal approach enabled us to sequentially add groups of predictor variables that reflected domains guided by the Family Health Development theory. To address scale differences in the observed indicators, all continuous independent variables were standardized to a z-scale, with a mean of zero and a standard deviation of one [58]. Control variables were included in final regression models. Multiple imputation was used to handle missing data and included sampling weights. All analyses were conducted in Stata/SE 18. Unstandardized and standardized coefficients are reported for OLS models. Odds ratios are reported for the binary logistic regression models.

## 4. Results

The first research question sought to determine participants’ mean depression and anxious personality scores, which are presented in Table 2. In total, 350 participants completed the 5-item CES-D depression scale at Wave IV, with a mean CES-D score of 3.13 (*SD* = 2.85). In total, 351 participants completed the anxious personality scale, with a mean score of 12.16 (*SD* = 2.87). The second research question focused on identifying the proportion of participants with depression and/or anxiety/panic disorder diagnoses. In total, 351 participants indicated whether they had or had not received a depression diagnosis or an anxiety/panic disorder diagnosis at Wave IV. About 20% of participants (*n* = 70) self-reported ever having a depression diagnosis, and about 12% of participants (*n* = 42) reported ever having an anxiety/panic disorder diagnosis. Only 8% of participants (*n* = 29) reported experiencing both depression and anxiety/panic disorder diagnoses.

### 4.1. Differences and Associations in Mental Health Symptoms

The third research question sought to determine differences and associations in mental health outcomes (i.e., depression scores, anxious personality scores) based on family structure, family process, cognitions, health behaviors, and demographic characteristics.

#### 4.1.1. Depression Scores

Using OLS with a hierarchical approach, scores from the CES-D depression scale were individually regressed on five models. See Table 3 for a summary of findings.

Overall, 14% of the variance in depression can be explained by the model (R^2^ = 0.14; Adj R^2^ = 0.10). Results indicate that belonging to a family receiving public assistance (β = 0.19, *p* ≤ 0.001), family belonging (β = −0.17, *p* ≤ 0.05), receiving mental health treatment (β = 0.12, *p* ≤ 0.05), engaging in exercise (β = −0.11, *p* ≤ 0.05), and trouble relaxing (β = 0.11, *p* ≤ 0.05) were significant predictors of depression symptoms. Those who were part of a family that received public assistance in adolescence had higher levels of depression symptoms, compared to those in families who did not receive public assistance. For every one-unit increase in a sense of family belonging depression scores decreased by 0.17 units, holding everything else constant. Those who engaged in mental health treatment, compared to those who had not, had significantly higher levels of depression symptoms. Those who had engaged in exercise during the prior week at Wave I reported decreased depression symptoms compared to those who had not exercised. Individuals who had trouble relaxing during adolescence had higher levels of depression, compared to those who did not have trouble relaxing. For every one-unit increase in cognitive ability, depression scores decreased by 0.11 units, holding everything else constant, though this result did not reach statistical significance (*p* = 0.07).

#### 4.1.2. Anxious Personality Scores

A hierarchal approach using OLS regressed anxious personality scores on five models that included four health domains representing the theoretical framework and control variables. See Table 4 for a summary of findings. Overall, 13% of the variance in anxious personality scores can be explained by the model (R^2^ = 0.13; Adj R^2^ = 0.09). Results indicated identifying as female (β = 0.18, *p* ≤ 0.001), identifying as Latinx, American Indian or Native American, and Asian or Pacific Islander (β = −0.12, *p* ≤ 0.05), and family receiving public assistance (β = 0.15, *p* ≤ 0.05) were significant predictors of anxiety symptoms. Compared to males, those who identified as female had higher levels of anxious personality symptoms. Those who identified as Black, Latinx, American Indian or Native American, and Asian or Pacific Islander had significantly lower levels of anxious personality symptoms compared to their white counterparts. Belonging to a family who received public assistance during adolescence, compared to a family that did not, resulted in higher levels of anxious symptoms. 

### 4.2. Differences and Associations in Mental Health Diagnoses

The final research question examined differences and associations in ever being diagnosed with depression or anxiety/panic disorder based on family structure, family process, cognitions, health behaviors, and demographic characteristics.

#### 4.2.1. Depression Diagnosis

Results from the binary logistic regressions using a hierarchal approach indicated six variables predicted having ever been diagnosed with depression (see Table 5). Females had 1.9 times higher odds of a depression diagnosis compared to male counterparts. Black participants had 0.34 times the odds of being diagnosed with depression compared to white individuals. Latinx, American Indian or Native American, and Asian or Pacific Islander participants had significantly lower odds of being diagnosed with depression compared to white participants. For every one-unit increase in depression symptoms, holding everything else constant, the odds of a depression diagnosis increased by 67%. The odds ratio for health knowledge on drug abuse indicates that participants who received health information related to drug abuse, compared to those who did not, had decreased odds of a depression diagnosis. The odds ratio for mental health treatment receipt shows that participants who received mental health treatment during adolescence, compared to counterparts who did not, had nearly 2.5 times higher odds of having a depression diagnosis.

#### 4.2.2. Anxiety/Panic Disorder Diagnosis

Results from the binary logistic regression with a hierarchical approach indicated that gender, race, depression scores during adolescence, health knowledge on drug abuse, and requiring but not receiving medical treatment were significantly associated with having an anxiety/panic disorder diagnosis (see Table 6).

Female participants were 2.61 times more likely to have a diagnosis of anxiety/panic disorder compared to males. Individuals who identified as Black had decreased odds of being diagnosed with anxiety/panic disorder compared to white participants. Similarly, individuals who identified as Latinx, American Indian or Native American, and Asian or Pacific Islander had even lower odds of being diagnosed with anxiety/panic disorder compared to white participants. Each unit increase in depression scores during adolescence, holding everything else constant, was associated with having 2.22 times higher odds of having an anxiety/panic disorder diagnosis.

Health knowledge on drug abuse was a significant negative predictor of anxiety/panic disorder diagnosis. Participants who received health knowledge on drug abuse had 0.18 times the odds of being diagnosed with anxiety/panic disorder compared to those who had not. Needing but not receiving medical treatment in adolescence was a significant positive predictor of anxiety/panic disorder diagnosis. Those who needed but did not receive medical treatment in adolescence had 2.79 times the odds of being diagnosed with anxiety/panic disorder, compared to counterparts. Receiving health knowledge on fitness was marginally associated (*p* = 0.07) with having an anxiety/panic disorder diagnosis. Finally, having trouble relaxing in adolescence was marginally associated (*p* = 0.07) with having an anxiety/panic disorder diagnosis.

## 5. Discussion

This study builds on prior research investigating long-term mental health consequences associated with CSE. To our knowledge, this is the first study to apply the Family Health Development theoretical framework [20] to explore associations between family structure, family processes, cognitions, and health-behaviors with depression as well as anxiety among young adults with CSE histories. We used a combination of scales and self-report items to gain a more comprehensive understanding of mental health outcomes.

### 5.1. Associations Between Social Identity and CSE on Mental Health

Consistent with the literature on social identity and CSE, our findings support associations between gender, race/ethnicity, and mental health outcomes. Concerning gender, women were twice as likely to have a depression diagnosis compared to men, aligning with the literature illustrating gendered patterns in adverse mental health outcomes for those affected by CSE [15,57]. While our findings indicate that women may be more likely to have increased anxious personality scores and receive a depression or anxiety diagnosis, this does not mean that men or gender-diverse populations are more immune to depression or anxiety [58]. Within the CSE literature, boys/men are often under-identified and under-served [8] due to stigma, the construction of gender norms, and the subsequent concealment of CSE [59,60]. Further, structural barriers to receiving mental health treatment range from specialty services (e.g., human trafficking courts) largely geared toward cis girls [61] to CSE-impacted males not presenting in traditional healthcare systems [62]. Building upon this, it is important to consider the mismatch between reported depression symptoms and diagnosis among women. While depression symptoms did not significantly vary by gender, women were almost twice as likely to have a depression diagnosis compared to men. This finding may also reflect women’s increased access and willingness to engage in mental health treatment compared to male counterparts.

Those who identified as Black, Latinx, American Indian or Native American, and Asian or Pacific Islander reported significantly lower levels of anxious personality symptoms and were significantly less likely to be diagnosed with depression or an anxiety/panic disorder compared to white counterparts. This reflects disparities in the general population in which Black adults report lower rates of depression symptoms compared to white adults but were more likely to receive a diagnosis when they did seek care [63,64]. From a structural perspective, it is critical that we consider issues in measurement tools, cultural stigma, and access to diagnosis and treatment, in addition to historic and systematic factors. The disproportionate policing and incarceration of people of color in the U.S. may be compounded by historic and contemporary practice of arresting CSE-impacted groups on prostitution-related charges [44,65]. Moreover, individuals may have perceptions of mental health treatment being coercive or fraught if they have previously been systems-involved [66], leading to delays in seeking treatment until clinical thresholds are met [67].

Research suggests that individuals who identify as female and Black, Indigenous, or from other racial or ethnic minority groups may exhibit greater resilience. Specifically, chronic hardship may produce more stress [68], which, in turn, provides opportunities to promote resilience [69] that can buffer against severe symptomology. Building upon this, our findings indicate that depression in adolescence, alongside identifying as female and Black, Latinx, American Indian or Native American, and Asian or Pacific Islander, is a significant predictor of anxiety and depression in young adulthood, reflecting the complex relationship between chronic stress and resilience. This highlights how distinct forms of intersecting oppression operate within CSE-impacted individuals to navigate the bounds of mental health recovery, which is closely tied to narrow constructions of “victimhood” that is largely white and female. Deconstructing “victimhood” requires a centering of historically marginalized groups [70] so that culturally responsive pathways to mental healthcare are further developed.

### 5.2. Family Health Development Model

Our findings showed that multiple familial-level factors during adolescence can help explain anxiety and depression outcomes at a later life stage.

#### 5.2.1. Family Structure

The significant association between growing up in a family that received public assistance during adolescence and experiencing depression and anxiety symptoms in young adulthood among CSE-impacted individuals aligns with the existing literature. For instance, financial hardship and low socioeconomic status are common characteristics among CSE-impacted individuals [8,71], and low socioeconomic status is a well-established risk factor for depression and anxiety [72,73]. However, the relationship between poverty and CSE is complex. While acute poverty and material need can increase vulnerability to CSE, economic disadvantage is often only one factor among many intersecting factors that increase risk, such as growing up in households that normalize the hyper-sexualization of children or involve intergenerational sex trading [8]. While poverty can be a precursor to CSE, it may also emerge or persist as a consequence of CSE-related trauma and disrupted developmental trajectories [74].

Families who rely on public assistance often experience broader structural disadvantages and pernicious social factors [75] which can erode one’s ability to cope with additional sources of adversity, thereby increasing vulnerability to depression or anxiety [72]. In addition, these families may face systemic barriers to accessing high-quality mental healthcare, including lack of available culturally responsive providers, insurance, and reliable transportation. These gaps can exacerbate the long-term psychological impact of both poverty and CSE. The long-term effects of poverty, such as housing instability and food insecurity, further compound distress across the lifespan. Moreover, many individuals who experience CSE during childhood remain in low-income conditions into adulthood [74], perpetuating intergenerational cycles of disadvantage that continue to erode mental health and limit access to effective mental health treatment and support.

#### 5.2.2. Family Processes

Our findings that family belonging is associated with reduced depressive symptoms aligns with prior research that identifies family relationships as a protective factor against depression [76] and a central component in building resilience [77]. Future research should investigate the role of family belonging in supporting CSE-impacted groups through their recovery process and other behavioral health issues, such as problematic substance use.

The quality of relationships between children and parents during adolescence was not significantly associated with mental health outcomes in adulthood for these respondents. Given that Wave I and IV data were collected approximately 12 years apart, we posit that changes in the quality of family relationships over time may have had a more significant impact on mental health outcomes than the quality of relationships at any single point in adolescence. The dynamic nature of family interactions during adolescence and young adulthood, whether marked by improvement or deterioration, could have influenced the respondents’ mental health trajectories more profoundly than static assessments. Additionally, intervening factors such as social support, coping strategies, and life events over the 12-year period may have played a critical role in shaping long-term mental health. This suggests that the evolution of family relationships, rather than their quality at a fixed moment in time, may be a more crucial predictor of adult mental health outcomes and should be examined in future research.

#### 5.2.3. Cognitions

To our knowledge, this is the first study to explore how cognitive skills and health knowledge, key components of health literacy, relate to mental health outcomes among CSE-impacted young men and women. In this context, health literacy refers to an individual’s capacity to obtain, process, and understand basic health information needed to make informed health-related decisions. Cognitive ability, measured using scores from the picture vocabulary test as a proxy for IQ, was not found to be associated with mental health symptoms or diagnoses for those impacted by CSE. These null findings highlight a gap in the current literature on CSE, where cognitive skills are rarely examined in relation to mental health. Future research should explore whether and how specific cognitive processes, such as decision-making, interact with trauma exposure and access to care to shape mental health trajectories for this population.

Our findings show that CSE-impacted individuals had significantly decreased odds of having a depression, anxiety, or panic disorder diagnosis when receiving education on drug abuse. This finding underscores the importance of early access to health information as a critical dimension of health literacy. Health information, especially when delivered during formative years, may equip individuals with tools to understand risk behaviors, seek help, and adopt protective strategies, which, taken together, may buffer against negative mental health issues. While substance use is an important behavioral health outcome for CSE-impacted individuals, our study focuses on how health knowledge, rather than the behavior itself, is linked to improved mental health trajectories. Future research should explore how other dimensions of health knowledge and literacy may influence one’s mental health in the context of CSE.

#### 5.2.4. Health-Related Behaviors

Those who had engaged in exercise had decreased depression symptoms while respondents who had trouble relaxing during adolescence had higher levels of depression, compared to their counterparts. Our findings align with research that consistently shows that exercise has a positive impact on mental health, including reducing symptoms of depression. Similarly, research suggests that among children who experienced sexual abuse, depression is associated with a lack of physical activity [78], and this appears to hold true for those who experience CSE. Further, engaging in exercise contributes to feelings of relaxation and relief [78]. Thus, it may be critical that physical exercise activities be implemented in interventions designed to reduce depressive symptoms among CSE-impacted groups.

Those who received mental health treatment during adolescence, compared to counterparts who did not, had higher odds of having a depression diagnosis. We did not know the timepoint in which respondents had the diagnosis; therefore, these may have been during the same life stage. However, it is also possible that some individuals who received treatment during adolescence experienced the onset of depression later in life, after the initial treatment period. The relationship between adolescent mental health treatment and later depression diagnoses could be influenced by various factors, including the nature and duration of the treatment, the severity of symptoms, and other life stressors that occurred in adulthood. It is important to consider that receiving treatment during adolescence may reflect a higher level of vulnerability to mental health issues, which could carry over into adulthood. Further investigation into the timing and continuity of mental healthcare and its impact over time is needed to better understand these associations.

Needing but not receiving medical treatment in adolescence was a significant positive predictor of anxiety/panic disorder diagnosis. This lack of treatment may lead to the chronic exacerbation of symptoms, as individuals do not have access to providers or interventions that could help manage their health-related needs. Additionally, the absence of professional support may impair the development of effective coping mechanisms, leaving individuals more vulnerable to experiencing heightened levels of distress and dysfunction. Consequently, not receiving necessary medical treatment during adolescence can have long-lasting effects, influencing mental health outcomes in adulthood.

### 5.3. Limitations

While this study provides important insights, there are several limitations that should be considered. The multiple linear regression models accounted for 14% and 13% of variance, respectively, and do not support causal inferences. Therefore, the findings from this study should be interpreted with caution. The CSE item measured if adolescents traded sex for drugs or money, which provides a severely limited perspective of sexual exploitation. Adolescents who were exploited for other items of value, such as the fulfillment of basic needs, were not captured. Additionally, this study used a school-based approach with a community-based sample which excludes adolescents who were part of the juvenile legal system or those who may have been suspended, expelled, or truant. Taken together, CSE may be underreported and underestimated in the Add Health dataset.

Our findings are limited by the nature of the available sample and variables. All adolescents in this study identified as cis boys or girls and were enrolled in school at Wave I. Therefore, findings are not representative of all adolescents in this age group, particularly those who identify as transgender, nonbinary, or other gender expansive identities and/or were not enrolled in school. Predictor and outcome variables in these analyses were self-reported and represent a static, cross-sectional view taken from Waves I and IV. Two of the outcome variables (i.e., depression and anxiety/panic disorder diagnoses) were self-reported and asked participants to indicate if they ever received the diagnosis. Therefore, we cannot say for certain at what life stage (e.g., adolescence) these diagnoses were given.

### 5.4. Implications for Research

This study highlights the critical need for more research on CSE-impacted children’s health outcomes at later life stages. To our knowledge, no available literature exclusively focuses on the behavioral or physical health outcomes of middle-age or older adults who were exploited during childhood. As CSE-impacted children transition through adulthood, it is imperative that we gain a more comprehensive understanding of their long-term health needs and identity strategies that can provide support over time. Using frameworks like Life Course Theory and Family Health Development can guide these studies. Researchers should use mixed methods designs and community-engaged research approaches to both gain access to an otherwise hard-to-reach group and help ensure that studies do not unintentionally retraumatize these individuals who may experience heightened structural vulnerabilities.

### 5.5. Implications for Practice

These findings call attention to the need for children and adults who have confirmed CSE histories to receive mental health screenings as part of a comprehensive model of care. Service providers should receive ongoing human trafficking training to ensure they feel equipped to understand the potentially unique experiences of cis boys and men, gender expansive individuals, and those who identify as Black, Latinx, American Indian or Native American, and Asian or Pacific Islander. Given the long-term effects of CSE, it is imperative that these individuals, regardless of age of CSE onset or time since exiting the exploitation, be provided with the appropriate health treatment and resources to support their long-term physical and behavioral health and well-being as they transition into and throughout adulthood. As such, health providers across settings should increase their capacity to serve this population.

## 6. Conclusions

This study provides valuable insights into the mental health outcomes of young adults affected by CSE during adolescence. Findings underscore the significant associations between various factors, such as gender, race/ethnicity, family structure, family processes, and health behaviors, with depression and anxiety in young adulthood. Moreover, the study highlights the importance of addressing disparities in mental health diagnoses, particularly among marginalized racial and ethnic groups, and the need for tailored interventions that consider the unique experiences of those affected by CSE. Although the study is limited by certain methodological constraints, its implications for both research and practice are clear. It is crucial to continue exploring the long-term mental health needs of CSE-impacted individuals and ensure that service providers are equipped with the tools and knowledge to offer appropriate, culturally responsive care.

## Figures and Tables

**Table 1:** Characteristics of CSE-impacted adolescents at Wave I (*N* = 502).

Variable	*M* (*SD*) or *n* (%)
Age	15.03 (1.34)
Gender	
Female	164 (32.67%)
Male	338 (67.33%)
Race	
White	250 (50.20%)
Black	149 (29.92%)
Latinx, American Indian/Native American, Asian or Pacific Islander	99 (19.88%)
Depression symptoms Wave 1	3.28 (3.09%)
Family Structure	
Two-parent household	
No	285 (56.77%)
Yes	217 (43.23%)
Receiving public assistance	
No	364 (83.87%)
Yes	70 (16.13%)
Family Processes	
Mother–child relationship	4.34 (0.70)
Father–child relationship	4.12 (0.79)
Family belonging	3.63 (0.90)
Cognitions	
Picture vocabulary test	95.04 (14.99)
Drug abuse education	
No	44 (8.80%)
Yes	456 (91.20%)
Exercise education	
No	43 (8.62%)
Yes	456 (91.38%)
Heath-related Behaviors	
Receipt of health treatment	
No	382 (76.40%)
Yes	118 (23.60%)
Receipt of mental health treatment	
No	385 (77.31%)
Yes	113 (22.69%)
Engaged in exercise	
No	92 (18.36%)
Yes	409 (81.64%)
Trouble relaxing	
No	264 (52.69%)
Yes	237 (47.31%)
Substance use	
No	150 (29.88%)
Yes	352 (70.12%)

**Table 2:** Descriptive statistics for mental health outcomes at Wave IV (*N* = 351).

Variable	*M* (*SD*) or *n* (%)
Age at Wave IV	27.99 (1.44)
Depression symptoms	3.13 (2.85)
Anxious personality symptoms	12.16 (2.87)
Depression diagnosis ever	
No	281 (80.06%)
Yes	70 (19.94%)
Anxiety or panic disorder ever	
No	309 (88.03%)
Yes	42 (11.97%)

**Table 3:** Summary of linear regression analyses predicting depression symptoms (*N* = 350).

	Model 1		Model 2		Model 3		Model 4		Model 5	
Independent Variable	*b* (*SE*)	*b*	*b* (*SE*)	*b*	*b* (*SE*)	*b*	*b* (*SE*)	*b*	*b* (*SE*)	*b*
Age	−0.14 (0.16)	−0.05	−0.11 (0.16)	−0.04	−0.16 (0.16)	−0.06	−0.17 (0.16)	−0.06	−0.22 (0.16)	−0.08
Gender (female)	0.20 (0.32)	0.03	0.07 (0.39)	0.01	0.14 (0.33)	0.02	0.13 (0.33)	0.02	0.02 (0.32)	0.00
Race (White—ref.)										
Black	0.22 (0.35)	0.03	−0.05 (0.36)	−0.01	0.09 (0.37)	0.01	−0.12 (0.39)	−0.02	0.06 (0.39)	0.01
Latinx, American Indian/Native American, Asian/Pacific Islander	−0.41 (0.42)	−0.06	−0.53 (0.41)	−0.07	−0.45 (0.42)	−0.06	−0.70 (0.42)	−0.09	−0.42 (0.43)	−0.06
Depression Symptoms (Wave I)	0.60 (0.17) ***	0.20	0.56 (0.17) ***	0.18	0.44 (0.19) *	0.14	0.38 (0.19) *	0.19	0.19 (0.20)	0.06
Family Structure										
Two-parent household			−0.08 (0.32)	−0.01	−0.02 (0.33)	−0.00	−0.02 (0.33)	−0.00	0.19 (0.33)	0.03
Receiving public assistance			1.31 (0.43) **	0.17	1.28 (0.43) **	0.17	1.20 (0.44) **	0.16	1.49 (0.45) ***	0.19
Family Processes										
Mother–child relationship					0.12 (0.20)	0.04	0.13 (0.19)	0.04	0.21 (0.19)	0.07
Father–child relationship					0.01 (0.20)	0.00	0.02 (0.20)	010	0.07 (0.20)	0.02
Family belonging					−0.37 (0.22)	−0.12	−0.50 (0.23) *	−0.17	−0.53 (0.23) *	−0.17
Cognitions										
Picture vocabulary test							−0.36 (0.18) *	−0.11	−0.34 (0.18)	−0.11
Drug abuse education							−0.92 (0.65)	−0.08	−0.80 (0.65)	−0.07
Exercise education							0.04 (0.64)	0.00	0.00 (0.64)	0.00
Heath-related Behaviors										
Receipt of health treatment									−0.62 (0.36)	−0.02
Receipt of mental health treatment									−0.79 (0.39) *	−0.01
Exercise									−0.86 (0.39) *	−0.02
Trouble relaxing									0.63 (0.32) *	0.10
Substance use									−0.15 (0.37)	−0.15
Model Fit										
Adj. *R*^2^	0.0315		0.0544		0.0556		0.0670		0.0981	
Model likelihood ratio *χ*^2^ (df, *p*-value)	3.27 (5, 0.0001)		5.18 (2, 0.0001)		1.14 (3, 0.0001)		2.39 (3, 0.0001)		3.31 (5, 0.0001)	

Note. Gender was coded: 0 = male, 1 = female. The reference group for race was white. * *p* < 0.05, ** *p* < 0.01, *** *p* < 0.0001.

**Table 4:** Summary of linear regression analyses predicting anxious personality symptoms (*N* = 350).

	Model 1		Model 2		Model 3		Model 4		Model 5	
Independent Variable	*b* (*SE*)	*b*	*b* (*SE*)	*b*	*b* (*SE*)	*b*	*b* (*SE*)	*b*	*b* (*SE*)	*b*
Age	−0.30 (0.15) *	−0.10	−0.28 (0.15)	−0.10	−0.30 (0.16)	−0.10	−0.30 (0.16)	−0.10	−0.32 (0.17)	−0.12
Gender (female)	1.32 (0.31) ***	0.31	1.24 (0.31) ***	0.21	1.18 (0.32) ***	0.10	1.15 (0.32) ***	0.20	1.08 (0.33) ***	0.18
Race (White—ref.)										
Black	−0.64 (0.34)	−0.10	−0.75 (0.35) *	−0.12	−0.75 (0.37) *	−0.12	−0.70 (0.39)	−0.11	−0.69 (0.40)	−0.11
Latinx, American Indian/Native American, Asian/Pacific Islander	−0.81 (0.40) *	−0.11	−0.88 (0.40) *	−0.12	−0.88 (0.41) *	−0.12	−0.95 (0.42) *	−0.13	−0.94 (0.43) *	−0.12
Depression Symptoms (Wave I)	0.38 (0.17) *	0.12	0.36 (0.17) *	0.11	0.29 (0.19)	0.09	0.28 (0.19)	0.09	0.24 (0.20)	0.08
Family Structure										
Two-parent household			−0.03 (0.31)	−0.01	0.03 (0.32)	0.01	0.05 (0.32)	0.01	0.08 (0.33)	0.01
Receiving public assistance			0.87 (0.42) *	0.11	0.92 (0.43) *	0.12	0.99 (0.44) *	0.13	1.14 (0.46) **	0.15
Family Processes										
Mother–child relationship					−0.19 (0.19)	−0.06	−0.19 (0.19)	−0.06	−0.18(0.19)	−0.57
Father–child relationship					−0.04 (0.20)	−0.01	−0.02 (0.20)	−0.01	−0.02 (0.20)	−0.01
Family belonging					−0.01 (0.22)	−0.00	−0.05 (0.23)	−0.02	−0.05 (0.23)	−0.02
Cognitions										
Picture vocabulary test							0.04 (0.18)	0.01	0.05 (0.19)	0.02
Drug abuse education							−0.72 (0.66)	−0.06	−0.76 (0.66)	−0.07
Exercise education							0.15 (0.64)	0.01	0.13 (0.65)	0.01
Heath-related Behaviors										
Receipt of health treatment									−0.10 (0.36)	−0.02
Receipt of mental health treatment									−0.06 (0.39)	−0.01
Exercise									−0.12 (0.39)	−0.02
Trouble relaxing									0.56 (0.32)	0.10
Substance use									−0.09 (0.38)	−0.01
Model Fit										
Adj. *R*^2^	0.10		0.10		0.10		0.09		0.09	
Model likelihood ratio *χ*^2^ (df, *p*-value)	8.11 (5, 0.0001)		2.30 (2, 0.0001)		0.48 (3, 0.0001)		0.42 (3, 0.0001)		0.61 (5, 0.0001)	

Note. Gender was coded: 0 = male, 1 = female. The reference group for race was white. * *p* < 0.05, ** *p* < 0.01, *** *p* < 0.0001.

**Table 5:** Summary of binary logistic regression analyses predicting a depression diagnosis (*N* = 350).

	Model 1			Model 2			Model 3			Model 4			Model 5		
Independent Variable	*OR*	*SE*	[95% CI]	*OR*	*SE*	[95% CI]	*OR*	*SE*	[95% CI]	*OR*	*SE*	[95% CI]	*OR*	*SE*	[95% CI]
Demographics															
Age	0.86	0.13	[0.64–1.15]	0.84	0.13	[0.63, 1.13]	0.81	0.13	[0.60, 1.10]	0.82	0.13	[0.60, 1.12]	0.74	0.13	[0.53, 1.04]
Gender (female)	1.86 *	0.55	[1.04, 3.32]	1.80	0.54	[1.00, 3.25]	1.98 *	0.62	[1.07, 3.66]	1.92 *	0.61	[1.03, 3.59]	1.93 *	0.64	[1.01, 3.70]
Race (White—ref.)															
Black	0.38 *	0.14	[0.19, 0.78]	0.29 *	0.11	[0.13, 0.61]	0.29 *	0.11	[0.13, 0.63]	0.27 *	0.11	[0.12, 0.61]	0.34 **	0.15	[0.14, 0.80]
Latinx, American Indian/Native American, Asian/Pacific Islander	0.32 *	0.15	[0.13, 0.80]	0.28 *	0.23	[0.11, 0.71]	0.29 *	0.14	[0.11, 0.75]	0.20*	0.10	[0.07, 0.55]	0.24 **	0.13	[0.08, 0.71]
Depression Symptoms (Wave I)	2.08 *	0.33	[1.53, 2.83]	2.02 *	0.31	[1.49, 2.74]	1.89 *	0.33	[1.35, 2.66]	1.92 *	0.34	[1.35, 2.73]	1.68 **	0.32	[1.16, 2.43]
Family Structure															
Two-parent household				0.47 *	0.15	[0.25, 0.88]	0.48 *	0.16	[0.25, 0.92]	0.48 *	0.16	[0.25, 0.93]	0.62	0.22	[0.31, 1.24]
Receiving public assistance				1.35	0.52	[0.63, 2.89]	1.26	0.50	[0.58, 2.74]	1.45	0.61	[0.64, 3.30]	1.92	0.87	[0.79, 4.66]
Family Processes															
Mother–child relationship							1.26	0.23	[0.88, 1.79]	1.26	0.23	[0.87, 1.81]	1.34	0.25	[0.93, 1.94]
Father–child relationship							0.90	0.17	[0.63, 1.29]	1.00	0.19	[0.69, 1.46]	1.11	0.22	[0.75, 1.65]
Family belonging							0.81	0.18	[0.52, 1.25]	0.68	0.16	[0.43, 1.08]	0.67	0.16	[0.42, 1.06]
Cognitions															
Picture vocabulary test										0.90	0.17	[0.63, 1.31]	0.92	0.18	[0.63, 1.36]
Drug abuse education										0.16 *	0.10	[0.05, 0.54]	0.15 **	0.09	[0.04, 0.51]
Exercise education										2.99	2.15	[0.73, 12.27]	2.73	2.02	[0.64, 11.64]
Health-related Behaviors															
Receipt of health treatment													1.44	0.50	[0.73, 2.86]
Receipt of mental health treatment													2.49 **	0.87	[1.25, 4.95]
Exercise													0.58	0.22	[0.27, 1.23]
Trouble relaxing													1.66	0.56	[0.86, 3.23]
Substance use													1.30	0.55	[0.57, 2.96]
McKelvey and Zavoina’s pseudo *R*^2^													0.37		
Model likelihood ratio *χ*^2^ (df, *p*-value)	44.57 (5, 0.0001)			51.89 (7, 0.0001)			54.45 (10, 0.0001)			64.91 (13, 0.0001)			79.31 (18, 0.0001)		

Note. Gender was coded: 0 = male, 1 = female. The reference group for race was white. * *p* < 0.05, ** *p* < 0.01.

**Table 6:** Summary of binary logistic regression analyses predicting an anxiety or panic disorder diagnosis (*N* = 351).

	Model 1			Model 2			Model 3			Model 4			Model 5		
Independent Variable	*OR*	*SE*	[95% CI]	*OR*	*SE*	[95% CI]	*OR*	*SE*	[95% CI]	*OR*	*SE*	[95% CI]	*OR*	*SE*	[95% CI]
Demographics															
Age	0.99	0.18	[0.68, 1.43]	0.97	0.18	[0.67, 1.40]	1.03	0.20	[0.70, 1.51]	1.03	0.20	[0.70, 1.52]	0.87	0.19	[0.59, 1.37]
Gender (female)	2.75 **	1.03	[1.32, 5.73]	2.78 **	1.05	[1.33, 5.84]	2.52 *	0.99	[1.17, 5.43]	2.53 **	1.01	[1.16, 5.52]	2.61	1.10	[1.14, 5.98]
Race															
Black	0.16 ***	0.09	[0.05, 0.48]	0.14 ***	0.08	[0.04, 0.44]	0.13 ***	0.07	[0.04, 0.40]	0.12 ***	0.07	[0.04, 0.39]	0.10 ***	0.07	[0.03, 0.39]
Latinx, American Indian/Native American, Asian/Pacific Islander	0.16 **	0.10	[0.04, 0.58]	0.15 **	0.10	[0.04, 0.56]	0.13 **	0.09	[0.03, 0.50]	0.09 ***	0.07	[0.02, 0.39]	0.07 ***	0.06	[0.02, 0.35]
Depression Symptoms (Wave I)	1.97	0.36	[1.37, 2.83]	1.94 ***	0.36	[1.35, 2.78]	2.21 ***	0.48	[1.44, 3.40]	2.27 ***	0.51	[1.46, 3.53]	2.22 ***	0.55	[1.37, 3.60]
Family Structure															
Two-parent household				0.56	0.21	[0.27, 1.19]	0.53	0.21	[0.24, 1.17]	0.53	0.22	[0.24, 1.18]	0.69	0.30	[0.29, 1.62]
Receiving public assistance				0.86	0.45	[0.31, 2.41]	0.96	0.51	[0.34, 2.71]	1.19	0.66	[0.40, 3.55]	1.96	1.20	[0.59, 6.52]
Family Processes															
Mother–child relationship							0.74	0.16	[0.49, 1.13]	0.72	0.16	[0.47, 1.11]	0.76	0.17	[0.50, 1.18]
Father–child relationship							1.02	0.23	[0.66, 1.59]	1.17	0.28	[0.74, 1.86]	1.26	0.31	[0.78, 2.06]
Family belonging							1.58	0.47	[0.89, 2.83]	1.37	0.42	[0.76, 2.48]	1.41	0.44	[0.76, 2.61]
Cognitions															
Picture vocabulary test										1.02	0.23	[0.65, 1.58]	0.94	0.22	[0.60, 1.48]
Drug abuse education										0.22	0.17	[0.05, 0.96]	0.18 *	0.14	[0.04, 0.86]
Exercise education										4.82	4.63	[0.73, 31.73]	6.60	6.91	[0.84, 51.49]
Health-related Behaviors															
Receipt of health treatment													2.79	1.19	[1.21, 6.44]
Receipt of mental health treatment													1.60 *	0.70	[0.68, 3.78]
Exercise													2.35	1.37	[0.75, 7.38]
Trouble relaxing													2.26	1.01	[0.94, 5.42]
Substance use													1.16	0.62	[0.40, 3.32]
McKelvey and Zavoina’s pseudo *R*^2^													0.52		
Model likelihood ratio *χ*^2^ (df, *p*-value)	46.63 (5, 0.0001)			48.97 (7, 0.0001)			52.44 (10, 0.0001)			57.48 (13, 0.0001)			72.50 (18, 0.0001)		

Note. Gender was coded: 0 = male, 1 = female. The reference group for race was white. * *p* < 0.05, ** *p* < 0.01, *** *p* < 0.0001.

## Data Availability

The original data presented in the study are openly available in the UNC Carolina Population Center at https://addhealth.cpc.unc.edu/data/ (accessed on 1 April 2024).

## Abbreviations

The following abbreviations are used in this manuscript:

ADHD	attention-deficit/hyperactivity disorder
CSE	commercial sexual exploitation
IQ	intelligence quotient
PTSD	posttraumatic stress disorder

## References

[1] Haney K., LeBeau K., Bodner S., Czizik A., Young M.E., Hart M. (2020). Sex trafficking in the United States: A scoping review. J. Evid. Based Soc. Work.

[2] Godoy S.M., Abrams L.S., Barnert E.S., Kelly M.A., Bath E.P. (2020). Fierce autonomy: How girls and young women impacted by commercial sexual exploitation perceive health and exercise agency in health care decision-making. Qual. Health Res..

[3] Ottisova L., Hemmings S., Howard L.M., Zimmerman C., Oram S. (2016). Prevalence and risk of violence and the mental, physical and sexual health problems associated with human trafficking: An updated systematic review. Epidemiol. Psychiatr. Sci..

[4] Simkhada P., van Teijlingen E., Sharma A., Bissell P., Poobalan A., Wasti S.P. (2018). Health consequences of sex trafficking: A systematic review. J. Manmohan Mem. Inst. Health Sci..

[5] Bath E., Barnert E., Godoy S., Hammond I., Mondals S., Farabee D., Grella C. (2020). Substance use, mental health, and child welfare profiles of juvenile justice-involved commercially sexually exploited youth. J. Child Adolesc. Psychopharmacol..

[6] Franchino-Olsen H., Chesworth B., Boyle C., Rizo C., Martin S., Jordan B., Macy R., Stevens L. (2022). The Prevalence of Sex Trafficking of Children and Adolescents in the United States: A Scoping Review. Trauma Violence Abus..

[7] Miller-Perrin C.L., Wurtele S.K., Bryce I., Robinson Y., Petherick W. (2017). What Works to Prevent the Sexual Exploitation of Children and Youth. The Wiley Handbook of What Works in Child Maltreatment: An Evidence-Based Approach to Assessment and Intervention in Child Protection.

[8] Godoy S.M., Hall W.J., Cal T., Bath E.P., Abrams L.S., Goode R.W., Jensen T., Chapman M.V. (2024). Children’s pathways into commercial sexual exploitation in the United States: A systematic review. Trauma Violence Abus..

[9] Barnert E., Kelly M., Godoy S., Abrams L.S., Rasch M., Bath E. (2019). Understanding commercially sexually exploited young women’s access to, utilization of, and engagement in health care: “Work around what I need”. Womens Health Issues.

[10] Bath E.P., Godoy S.M., Morris T.C., Hammond I., Mondal S., Goitom S., Barnert E.S. (2020). A specialty court for US youth impacted by commercial sexual exploitation. Child Abuse Negl..

[11] Chapple C.L., Crawford B.L. (2019). Mental health diagnoses of youth commercial sex exploitation victims: An analysis within an adjudicated delinquent sample. J. Fam. Viol..

[12] Le P.D., Ryan N., Rosenstock Y., Goldmann E. (2018). Health issues associated with commercial sexual exploitation and sex trafficking of children in the United States: A systematic review. Behav. Med..

[13] Lederer L.J., Wetzel C.A. (2014). The health consequences of sex trafficking and their implications for identifying victims in healthcare facilities. Ann. Health Law.

[14] Palines P.A., Rabbitt A.L., Pan A.Y., Nugent M.L., Ehrman W.G. (2020). Comparing mental health disorders among sex trafficked children and three groups of youth at high-risk for trafficking: A dual retrospective cohort and scoping review. Child Abuse Negl..

[15] Hossain M., Zimmerman C., Abas M., Light M., Watts C. (2010). The relationship of trauma to mental disorders among trafficked and sexually exploited girls and women. Am. J. Public Health.

[16] Barnert E.S., Bath E., Heard-Garris N., Lee J., Guerrero A., Biely C., Dudovitz R. (2022). Commercial sexual exploitation during adolescence: A US-based national study of adolescent to adult health. Public Health Rep..

[17] Turgumbayev M., Rima D., Duzbayeva S., Alimova E., Beaver K.M. (2023). Long-term mental health, victimization, and behavioral consequences associated with human sex trafficking. Crime Law Soc. Change.

[18] Lanctot N., Turcotte M., Pascuzzo K., Collin-Vezina D., Laurier C. (2021). Commercial Sexual Exploitation, Stigma, and Trauma: A Detrimental Trio for an Altered Sense of Self. J. Child Sex. Abus..

[19] Cole J., Sprang G., Lee R., Cohen J. (2016). The Trauma of Commercial Sexual Exploitation of Youth: A Comparison of CSE Victims to Sexual Abuse Victims in a Clinical Sample. J. Interpers. Violence.

[20] Feinberg M., Hotez E., Roy K., Ledford C.J.W., Lewin A.B., Perez-Brena N., Childress S., Berge J.M. (2022). Family health development: A theoretical framework. Pediatrics.

[21] Halfon N., Hochstein M. (2002). Life course health development: An integrated framework for developing health, policy, and research. Milbank Q..

[22] Langton C.E., Berger L.M. (2011). Family Structure and Adolescent Physical Health, Behavior, and Emotional Well-Being. Soc. Serv. Rev..

[23] Mazzuco S., Meggiolaro S. (2014). Family Structures and Health Behaviour in Adolescents. Child Indic. Res..

[24] McGowan E.C., Hofheimer J.A., O’Shea M.T., Kilbride H., Carter B.S., Check J., Helderman J., Neal C.R., Pastyrnak S., Smith L.M. (2022). Analysis of Neonatal Neurobehavior and Developmental Outcomes Among Preterm Infants. JAMA Netw. Open.

[25] Wasserman M. (2020). The Disparate Effects of Family Structure. Future Child..

[26] Assari S., Caldwell C.H., Zimmerman M.A. (2018). Family Structure and Subsequent Anxiety Symptoms; Minorities’ Diminished Return. Brain Sci..

[27] Wu S., Fraser M.W., Chapman M.V., Gao Q., Huang J., Chowa G.A. (2018). Exploring the Relationship between Welfare Participation in Childhood and Depression in Adulthood in the United States. Soc. Sci. Res..

[28] Saewyc E.M., Edinburgh L.D. (2010). Restoring Healthy Developmental Trajectories for Sexually Exploited Young Runaway Girls: Fostering Protective Factors and Reducing Risk Behaviors. J. Adolesc. Health.

[29] Jensen T.M., Harris K.M. (2017). Stepfamily Relationship Quality and Stepchildren’s Depression in Adolescence and Adulthood. Emerg. Adulthood.

[30] Chaney D.W. (2013). An Overview of the First Use of the Terms Cognition and Behavior. Behav. Sci..

[31] Gershon R., Wagster M., Hendrie H., Fox N., Cook K., Nowinski C. (2013). NIH Toolbox for Assessment of Neurological and Behavioral Function. Neurology.

[32] Ek U., Westerlund J., Fernell E. (2013). General versus Executive Cognitive Ability in Pupils with ADHD and with Milder Attention Problems. Neuropsychiatr. Dis. Treat..

[33] Weeks M., Wild T.C., Ploubidis G.B., Naicker K., Cairney J., North C.R., Colman I. (2014). Childhood Cognitive Ability and Its Relationship with Anxiety and Depression in Adolescence. J. Affect. Disord..

[34] Ryan J., Jacob B., Gross M., Perron B., Moore A., Ferguson-Johnson S. (2018). Early Exposure to Child Maltreatment and Academic Outcomes. Child Maltreat..

[35] Vaillancourt A.A. (2019). Executive Functioning Profile of Adolescents with a History of Prostitution.

[36] Greenbaum V.J. (2014). Commercial Sexual Exploitation and Sex Trafficking of Children in the United States. Curr. Probl. Pediatr. Adolesc. Health Care.

[37] Moussavi S., Chatterji S., Verdes E., Tandon A., Patel V., Üstün B. (2007). Depression, Chronic Diseases, and Decrements in Health: Results from the World Health Surveys. Lancet.

[38] Lincoln A., Paasche-Orlow M.K., Cheng D.M., Lloyd-Travaglini C., Caruso C., Saitz R., Samet J.H. (2006). Impact of Health Literacy on Depressive Symptoms and Mental Health–Related Quality of Life among Adults with Addiction. J. Gen. Intern. Med..

[39] Bożek A., Nowak P., Blukacz M. (2020). The Relationship Between Spirituality, Health-Related Behavior, and Psychological Well-Being. Front. Psychol..

[40] Berendsen J., Bonifácio C., Gemert-Schriks M., Loveren C., Verrips E., Duijster D. (2018). Parents’ Willingness to Invest in Their Children’s Oral Health. J. Public Health Dent..

[41] Shi H.-J., Nakamura K., Takano T. (2004). Health Values and Health-Information-Seeking in Relation to Positive Change of Health Practice Among Middle-Aged Urban Men. Prev. Med..

[42] Naik A.D., Martin L.A., Moye J., Karel M.J. (2016). Health Values and Treatment Goals of Older, Multimorbid Adults Facing Life-Threatening Illness. J. Am. Geriatr. Soc..

[43] Wallace C., Lavina I., Mollen C. (2021). Share our stories: An exploration of the healthcare experiences of child sex trafficking survivors. Child Abuse Negl..

[44] Ijadi-Maghsoodi R., Bath E., Cook M., Textor L., Barnert E. (2018). Commercially sexually exploited youths’ health care experiences, barriers, and recommendations: A qualitative analysis. Child Abuse Negl..

[45] Hornor G., Sherfield J. (2018). Commercial sexual exploitation of children: Health care use and case characteristics. J. Pediatr. Health Care.

[46] Kawser M., Nazrul M., Khan I., Hossain J., Islam N. (2020). Socioeconomic, Behavioural and Sexual-Health Factors Associated with Nutritional Status of Female Commercial Sex Workers in Dhaka City, Bangladesh: A Cross-Sectional Study. Porto Biomed. J..

[47] Zhang Z., Jackson S.L., Gillespie C., Merritt R., Yang Q. (2023). Depressive Symptoms and Mortality Among US Adults. JAMA Netw. Open.

[48] Cyders M.A., Hunton T., Hershberger A.R. (2021). Substance Use and Childhood Sexual Abuse among Girls Who Are Victims of Commercial Sexual Exploitation. Subst. Use Misuse.

[49] Greenbaum V.J., Yun K., Todres J. (2018). Child trafficking: Issues for policy and practice. J. Law Med. Ethics.

[50] Harris K.M., Halpern C.T., Haberstick B.C., Smolen A. (2013). The National Longitudinal Study of Adolescent Health (Add Health) Sibling Pairs Data. Twin Res. Hum. Genet..

[51] Harris K.M., Halpern C.T., Whitsel E.A., Hussey J.M., Killeya-Jones L.A., Tabor J., Dean S.C. (2019). Cohort Profile: The National Longitudinal Study of Adolescent to Adult Health (Add Health). Int. J. Epidemiol..

[52] DeVellis R.F. (2017). Scale Development: Theory and Applications.

[53] Harris K.M., Ryan S., Day R.D., Lamb M.E. (2004). Father involvement and the diversity of family context. Conceptualizing and Measuring Father Involvement.

[54] Carolina Population Center National Longitudinal Study of Adolescent Health: Wave I Adolescent In-Home Questionnaire Code Book, Introductory Guides. https://www.cpc.unc.edu/projects/addhealth/documentation/restricteduse/datasets.

[55] Kahn N.F., Halpern C.T. (2018). The relationship between cognitive ability and experiences of vaginal, oral, and anal sex in the United States. J. Sex Res..

[56] Sinha P., Calfee C.S., Delucchi K.L. (2021). Practitioner’s guide to latent class analysis: Methodological considerations and common pitfalls. Crit. Care Med..

[57] Edinburgh L., Pape-Blabolil J., Harpin S.B., Saewyc E. (2015). Assessing Exploitation Experiences of Girls and Boys Seen at a Child Advocacy Center. Child Abuse Negl..

[58] Moynihan M., Mitchell K., Pitcher C., Havaei F., Ferguson M., Saewyc E. (2018). A Systematic Review of the State of the Literature on Sexually Exploited Boys Internationally. Child Abuse Negl..

[59] Forringer-Beal A. (2022). Why the ‘Ideal Victim’ Persists: Queering Representations of Victimhood in Human Trafficking Discourse. Anti-Trafficking Rev..

[60] Josenhans V., Kavenagh M., Smith S., Wekerle C. (2020). Gender, Rights and Responsibilities: The Need for a Global Analysis of the Sexual Exploitation of Boys. Child Abuse Negl..

[61] Godoy S.M., Perris G., Thelwell M., Osuna-Garcia A., Barnert E., Bacharach A., Bath E. (2022). A systematic review of specialty courts in the United States for adolescents impacted by commercial sexual exploitation. Trauma Violence Abus..

[62] Varma S., Gillespie S., McCracken C., Greenbaum V.J. (2015). Characteristics of Child Commercial Sexual Exploitation and Sex Trafficking Victims Presenting for Medical Care in the United States. Child Abuse Negl..

[63] Barnes D.M., Bates L.M. (2017). Do Racial Patterns in Psychological Distress Shed Light on the Black-White Depression Paradox? A Systematic Review. Soc. Psychiatry Psychiatr. Epidemiol..

[64] Hankerson S.H., Suite D., Bailey R.K. (2015). Treatment Disparities among African American Men with Depression: Implications for Clinical Practice. J. Health Care Poor Underserved.

[65] Abrams L., Godoy S.M., Bth E., Barnert E.S. (2020). Collaborative responses to commercial sexual exploitation as a model of smart decarceration. Soc. Work.

[66] Ijadi-Maghsoodi R., Cook M., Barnert E.S., Gaboian S., Bath E. (2016). Understanding and Responding to the Needs of Commercially Sexually Exploited Youth: Recommendations for the Mental Health Provider. Child Adolesc. Psychiatr. Clin. N. Am..

[67] Bailey R.K., Mokonogho J., Kumar A. (2019). Racial and Ethnic Differences in Depression: Current Perspectives. Neuropsychiatr. Dis. Treat..

[68] Williams D.R., Lawrence J.A., Davis B.A., Vu C. (2019). Understanding How Discrimination Can Affect Health. Health Serv. Res..

[69] Anderson L.A. (2019). Rethinking resilience theory in African American families: Fostering positive adaptations and transformative social justice. J. Fam. Theory Rev..

[70] Crenshaw K.W. (1991). Mapping the Margins: Intersectionality, Identity Politics, and Violence against Women of Color. Stan. Law Rev..

[71] Reid J.A., Baglivio M.T., Piquero A.R., Greenwald M.A., Epps N. (2019). No youth left behind to human trafficking: Exploring profiles of risk. Am. J. Orthopsychiatry.

[72] Lemstra M., Neudorf C., D’Arcy C., Kunst A., Warren L.M., Bennett N.R. (2008). A Systematic Review of Depressed Mood and Anxiety by SES in Youth Aged 10–15 Years. Can. J. Public Health.

[73] Lorant V., Deliège D., Eaton W., Robert A., Philippot P., Ansseau M. (2003). Socioeconomic Inequalities in Depression: A Meta-Analysis. Am. J. Epidemiol..

[74] Polaris (2023). Harm’s Way: How Systems Faily Human Trafficking Survivors.

[75] Adkins D.E., Wang V., Dupre M.E., van den Oord E.J.C.G., Elder G.H. (2009). Structure and Stress: Trajectories of Depressive Symptoms across Adolescence and Young Adulthood. Soc. Forces.

[76] Santini Z.I., Koyanagi A., Tyrovolas S., Mason C., Haro J.M. (2015). The association between social relationships and depression: A systematic review. J. Affect. Disord..

[77] MacPhee D., Lunkenheimer E., Riggs N. (2015). Resilience as regulation of developmental and family processes. Fam. Relat..

[78] Kendall-Tackett K. (2002). The Health Effects of Childhood Abuse: Four Pathways by Which Abuse Can Influence Health. Child Abus. Negl..

